# An undergraduate medical education framework for refugee and migrant health: Curriculum development and conceptual approaches

**DOI:** 10.1186/s12909-022-03413-8

**Published:** 2022-05-16

**Authors:** Douglas Gruner, Yael Feinberg, Maddie J. Venables, Syeda Shanza Hashmi, Ammar Saad, Douglas Archibald, Kevin Pottie

**Affiliations:** 1 Faculty of Medicine, Department of Family Medicine, University of Ottawa, Ottawa, ON Canada; 2 Department of Family Medicine, C.T. Lamont Primary Health Care Centre, University of Ottawa, Ottawa, ON Canada; 3 Department of Psychiatry, University of Toronto, Toronto, ON Canada; 4 School of Epidemiology and Public Health, University of Ottawa, Ottawa, ON Canada; 5 Bruyere Research Insitute, Ottawa, ON Canada; 6 Institut Savoir Montfort, Ottawa, ON Canada; 7 Family Medicine, Western University, London, ON Canada

**Keywords:** Refugees and migrants, Cultural competence, Cultural safety, Disease prevention, Social accountability, Undergraduate medical education framework

## Abstract

**Background:**

International migration, especially forced migration, highlights important medical training needs including cross-cultural communication, human rights, as well as global health competencies for physical and mental healthcare. This paper responds to the call for a ‘trauma informed’ refugee health curriculum framework from medical students and global health faculty.

**Methods:**

We used a mixed-methods approach to develop a guiding medical undergraduate refugee and migrant health curriculum framework. We conducted a scoping review, key informant interviews with global health faculty with follow-up e-surveys, and then, integrated our results into a competency-based curriculum framework with values and principles, learning objectives and curriculum delivery methods and evaluation.

**Results:**

The majority of our Canadian medical faculty respondents reported some refugee health learning objectives within their undergraduate medical curriculum. The most prevalent learning objective topics included access to care barriers, social determinants of health for refugees, cross-cultural communication skills, global health epidemiology, challenges and pitfalls of providing care and mental health. We proposed a curriculum framework that incorporates values and principles, competency-based learning objectives, curriculum delivery (i.e., community service learning), and evaluation methods.

**Conclusions:**

The results of this study informed the development of a curriculum framework that integrates cross-cultural communication skills, exploration of barriers towards accessing care for newcomers, and system approaches to improve refugee and migrant healthcare. Programs should also consider social determinants of health, community service learning and the development of links to community resettlement and refugee organizations.

**Supplementary Information:**

The online version contains supplementary material available at 10.1186/s12909-022-03413-8.

## Background

Cross-cultural education and global migration connect human rights, social development, evidence-based medicine and universal healthcare access [1, 2]. Indeed, the COVID-19 pandemic has magnified the health and social inequities facing refugee and migrant populations [3, 4]. Health inequities are unjust and unfair health disparities [5, 6]. Social accountability in medical education provides a compelling and promising case-by-case learning approach to disadvantaged populations [7]. However, medical students will require training opportunities in an interdisciplinary way to develop knowledge, skills and attitudes to address health inequities related to trauma, racism, culture and language differences, access to health systems, tropical infectious disease, vaccination, chronic disease and global mental health [8–10].

Refugee and migrant or newcomer health is a field of study that focuses on the health of forcibly displaced and migrating populations [11]. The International Organization of Migration estimates there are 272 million international migrants worldwide, 80 million of whom were forcibly displaced in 2019 [2]. Canada, for example, where 21% of the total population is foreign-born, resettles over 500,000 newcomers annually [12]. Transitioning refugee populations require evidence based clinical preventive guidelines[1, 13] community partnership programs[14–16] and innovative health systems [17]. Both physicians and newcomer patients routinely juggle multiple access to care barriers, acute and chronic disease epidemiology differences and language and health literacy challenges [18, 19].

Competency-based medical curriculum frameworks can guide the development of global health and social accountability curricula for both faculty and medical students. Existing frameworks include values and principles, teaching and learning methods, and core competency based learning objectives and evaluation [20, 21]. Competency based roles (i.e. CanMEDs roles: Expert, Communicator, Collaborator, Advocate, Professional, Leader, and Scholar)[22] sit at the centre of these frameworks and drive the learning objectives. Teaching and learning on trauma informed communication approaches, patient-centred and evidence-based interventions, mental health care and antiracism, and related advocacy[16, 23] are high priority topics in social accountability[24].

Skilled and well-prepared future physicians and leaders could reduce the unjust and unfair health disparities that face refugees and migrants [25–27]. Medical students have called for more innovative global and refugee health training approaches [28, 29]. The objective of this paper was to develop a practical framework to improve medical student curriculum that will enable the next generation of physicians to address refugee and migrant/newcomer health inequities.

## Methods

**Fig. 1:**
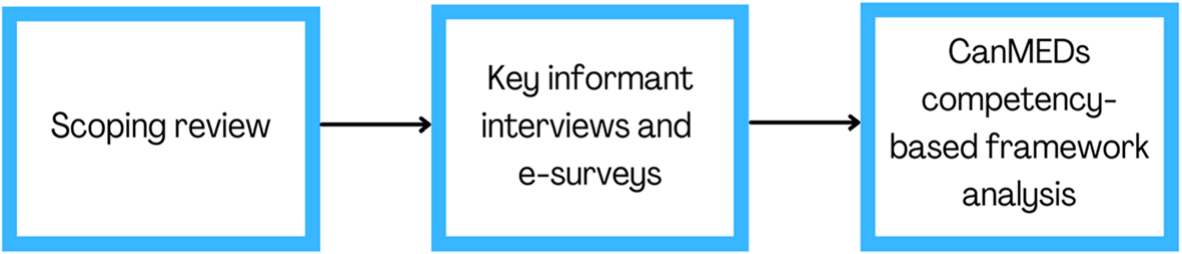
Logic Model of Refugee Health Curriculum Framework Mixed-Methods Development

We used a multi-phased mixed methods approach[30] to identify priority topics and develop new objectives and curriculum delivery methods for an undergraduate medical curriculum on refugee and migrant health (Fig. 1). We report our findings according to the Good Reporting of a Mixed Methods Study (GRAMMS) reporting guidelines [31].

We performed a scoping review, conducted key informant interviews with global health faculty, followed up with refugee health curriculum e-surveys, and finally, used a competency-based framework analysis to develop and refine priority learning objectives and curriculum delivery methods. Mixed methods have allowed us to identify elements of a refugee curriculum more comprehensively than any single method alone. We followed a collaborative research approach [32], and engaged key stakeholder groups, including medical students, refugee health experts, and people with lived experience of migration, in the conceptualization of our methods and interpretation and integration of our findings in the curriculum framework. This project was reviewed by the Ottawa Health Science Network Review and Ethics Board and approved as a quality improvement study.

### Phase 1: Scoping Review

The objective of our systematic scoping review was to identify and map published international educational content and interdisciplinary educational approaches that address medical undergraduate level refugee and migrant/newcomer health training. This scoping review also aimed to identify knowledge gaps regarding refugee and migrant health education at the undergraduate medical level. The methodology for this scoping review followed the approach developed by Arksey and O’Malley (2005) [33]. Findings were grouped by educational content, content delivery methods and educational outcomes. Detailed methods concerning our information sources, search strategy, record screening and extraction and data mapping and reporting are described in Additional file 1.

### Phase 2: a) Key Informant Interviews with Global Health Faculty

We conducted interviews from December 2018 to February 2019 with global health and medical faculty to identify existing curriculum initiatives and to explore perspectives regarding opportunities for refugee and newcomer health education at the undergraduate level. Using the Association of Faculties of Medicine of Canada Global Health contact list, we purposely selected key informants from 17 medical schools. Each key informant was invited to participate in a virtual interview (phase 2a) and an e-survey (phase 2b). A total of 13 interviews, ranging from 21–51 min in length were conducted with global health key informants representing 14 of the 17 undergraduate medical programs across Canada. Our interview guide (see Additional file 2) was informed by the results of our scoping review as well as input from refugee health experts on our team (DG,KP).

The semi-structured interviews were recorded using Zoom Video Communications [34]. Each interview was transcribed verbatim with the assistance of a online voice-recognition platform (Otter.ai) and manually reviewed by one of our team members (YF) to ensure transcription accuracy [35].

Two independent reviewers (HT, SSH, MV as the first reviewer, and YF as the second reviewer) analyzed the interview data and emerging themes using a “best-fit” framework analysis approach (using constructs from the Bierman migration framework model) [36, 37]. We selected the Bierman model as our theoretical framework because it incorporates elements of theory, the experience of migration and its intersection with social determinants of health, racial and ethnic disparities, and gender equity [36]. We identified and coded themes manually, sought outliers and developed an overall analysis, topics, methods and challenges.

### Phase 2: b) Refugee Health Curriculum e-Surveys

We used Survey Monkey to conduct e-surveys in order to descriptively obtain refugee curriculum data from our selected faculty key informants. We used topics from the scoping review and interviews to refine our survey questions and collected data from December 2018 to May 2019. We then used the tailored design method for conducting surveys to maximize our response rate, by sending key informants three separate reminders to complete the survey [38]. We descriptively analyzed demographics, dedicated undergraduate medical education curriculum hours, refugee and migrant health topics and learning (delivery) methods. We also identified existing undergraduate medical education refugee learning objectives (see Additional file 3 for survey questions).

### Phase 3: Developing a Refugee and Migrant Health competency-based framework

In an effort to build on related social accountability medical curriculum frameworks, our team reviewed and adopted existing values and principles on global health curricula development [21]. We then proceeded to integrate our emerging refugee and migrant health learning topics as learning objectives within CanMeds competencies (2017) and existing teaching methods to create a complementary refugee health curriculum framework. We followed the methods outlined in Hashmi et al*.* (2020), integrating learning objectives and teaching methods from the scoping, interview and e-survey results into a CanMEDs framework that includes refugee and migrant health. Two reviewers (SH,HT) systematically integrated our topics into curriculum objectives and teaching methods. Three reviewers (DG, KP, DA) challenged, verified and sought a consensus on the integrated curriculum objectives and methods. As a final step, to improve the reliability of our framework analysis, we brought the emerging values and principles, curriculum learning objectives and delivery methods back to four faculty key informants as a final member checking exercise.

## Results

Our mixed methods approach identified a series of values and principles, topics and teaching methods relevant for refugee and migrant health curriculum.

### Scoping Review

Our scoping review included a total of seventeen articles, describing educational topics (e.g., cultural safety and cross-cultural communication, disease screening and prevention) and interdisciplinary content delivery methods (e.g., experiential and community service learning). Please see Additional files 4 and 5 for detailed results of this review.

### Key Informant Interviews and e-Surveys

#### Description of Participants

A total of 13 anonymized key informants, representing faculty leads in global health and/or undergraduate refugee health medical education in their respective institutions, participated in the interviews and surveys.

#### Interviews

**Fig. 2:**
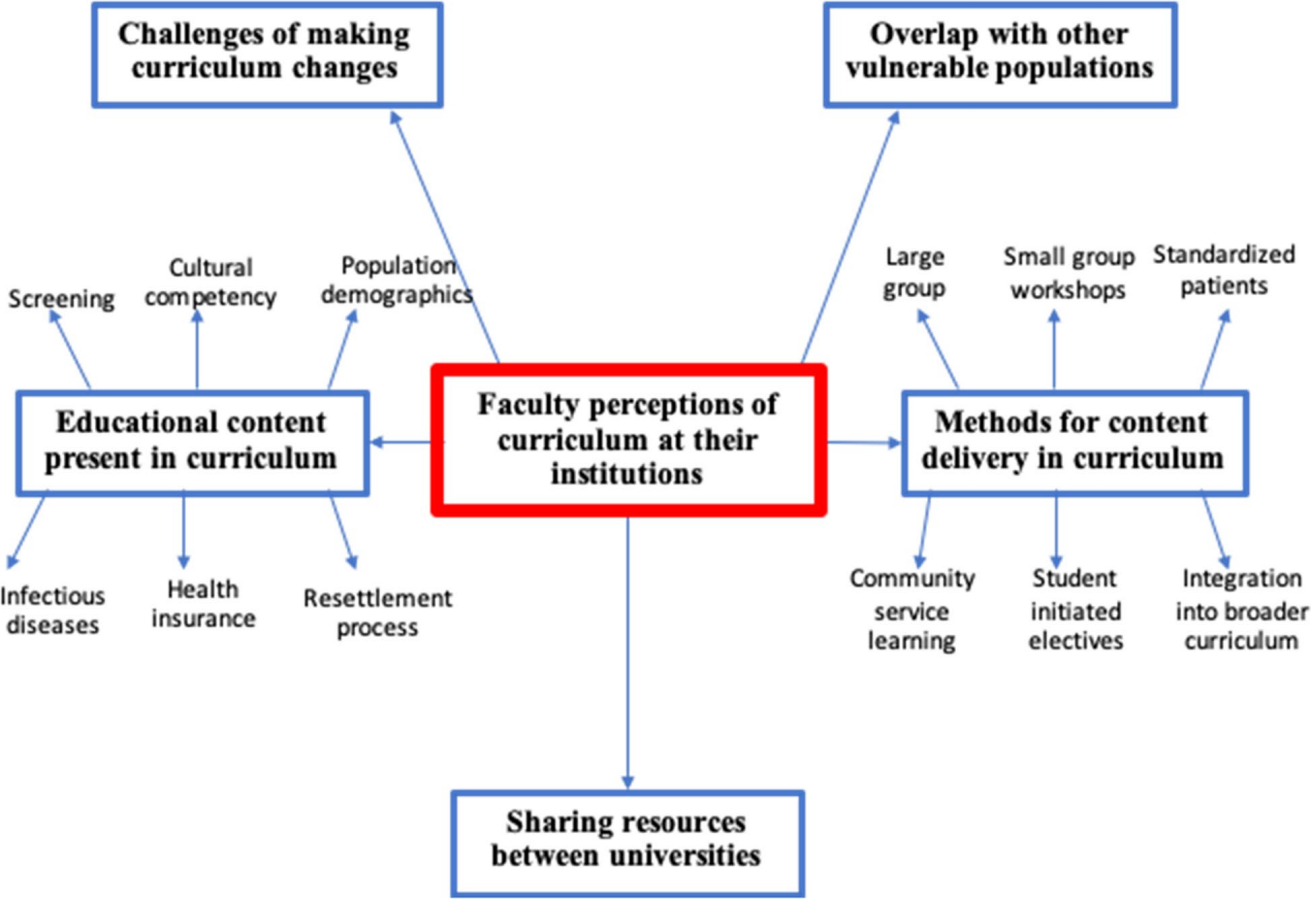
Emerging themes from interviews with faculty concerning undergraduate refugee health curriculum in Canada

The following five themes emerged from the interviews: recognizing existing specific refugee and migrant health learning objectives, active teaching methods, overlap with other underserved populations and social accountability education, challenges of implementing a refugee and migrant health curricula within undergraduate medical education, and the value of sharing educational resources across Canadian medical schools (see Fig. 2).

The majority of key informants reported that their undergraduate medical curricula covered topics such as the demographics of refugees and migrants, barriers faced when accessing health care, challenges of providing health care to migrants, settlement support services in the community, communications skills and cultural competency/safety, preventive care screening guidelines and social determinants of health.

Key informants spoke about the various methods of how they delivered the refugee health curriculum. Delivery methods included large group didactic sessions, panel discussions with different professionals, small group workshops with standardized patients, online modules, and independent readings. Most informants also reported community service-learning programs (e.g., pairing students with refugee families to help them acclimatize to Canada). Key informants also discussed partnering opportunities with settlement agencies for students to do observerships within primary care clinics and/or settlement housing, where they would be assessed on their ability to perform medical histories and intake assessments.

Several key informants mentioned that the majority of their refugee and migrant health content overlapped or was integrated with other underserved populations within the curriculum. For example, education sessions discussing topics such as social determinants of health or cultural competencies were also felt to be applicable to refugee and migrant populations but were sometimes discussed in the context of indigenous populations, those struggling with addictions and patients reporting unstable housing*.* Further refugee health content was often embedded in talks related to acute and chronic infectious diseases (i.e., Tuberculosis (TB), Human Immunodeficiency Virus (HIV), Hepatitis C Virus (HCV)) and other curriculum.

Challenges to implementing refugee health curricula included “limited faculty support”, specifically the small number of refugee health clinicians who are able to deliver the curriculum. Another challenge involved “time constraints within the curriculum”, so when advocating for curriculum change, balancing the overcrowded curriculum to ensure refugee health did not displace other underserved populations. Other key informants discussed difficulty regarding student engagement with non-mandatory learning topics that do not have significant weight on examinations.

Finally, several key informants expressed a desire to share existing refugee teaching resources and materials across Canadian medical schools. (See Additional file 6 for other significant quotes).

*“I would love to expand on refugee health teaching. Why start from scratch when things already exist. If other universities have good resources, I would be delighted to access and piggyback on those resources*.”- *Interview #3*

#### e-Surveys

A total of 14/17 survey responses were collected, representing a response rate of 82.4%. Please see Additional file 7 for details about the characteristics of survey responders. Survey responders affirmed the refugee-health educational content present in their various university curricula, as well as the methods by which content was delivered (See Tables 1 and 2).

Respondents reported a range of education methods used to deliver educational elements from their respective curricula. Methods included large group lectures, small group workshops, pre-clerkship experiences such as service-learning placements (i.e., partnering with settlement agencies), and clinical experiences during clerkship. Other tools included refugee health e-learning (see Table 2)

Additionally, over 40% (6/14) medical schools in Canada spend between 5–10 h during the entire undergraduate medical program delivering refugee/migrant health curriculum. Another 40% provide less than 5 h, and the remaining 2 schools spend 10–20 h over the entire undergraduate program

We collated the key (primary and secondary) learning topics from the scoping review, the interviews, and the surveys in Tables 3 and 4

We adopted existing values and principles from Redwood-Campbell et al*.*, (2011) (See Table 5).

### Proposed Curriculum Framework

We reviewed and debated the emerging key topics, learning objectives and educational delivery and evaluation methods. Using the CanMEDS Family Medicine competency framework we created a list of competencies integral towards providing care and addressing health inequities for refugee and migrant patients. After consensus within the team, a set of unique refugee health competencies emerged which are outlined in Table 6.

## Discussion

The COVID-19 pandemic has highlighted and often magnified the health and social inequities that refugee and migrant populations face [39]. Coinciding with this reality, medical students have expressed a desire to work with refugees and to address the needs of migrant populations. Refugees show great resilience and thus may provide important learning opportunities for medical students. Our proposed undergraduate medical curriculum framework for refugee and migrant health provides students, faculty, and medical schools with a practical guide to enhance their competency-based current curriculum development and approach.

Our mixed methods sequential approach allowed us to explicitly identify and build on existing curriculum topics, values, and resources. Indeed, we were able to bring these curriculum topics to our interviews and to therefore collect real world experience from Canadian medical schools on existing curriculum. Finally, the richness of our findings allowed us to ultimately bring together learning objectives, teaching methods and evaluation. Our weakest findings related to curriculum evaluation methods, and we suggest more research is needed in this area.

**Fig. 3:**
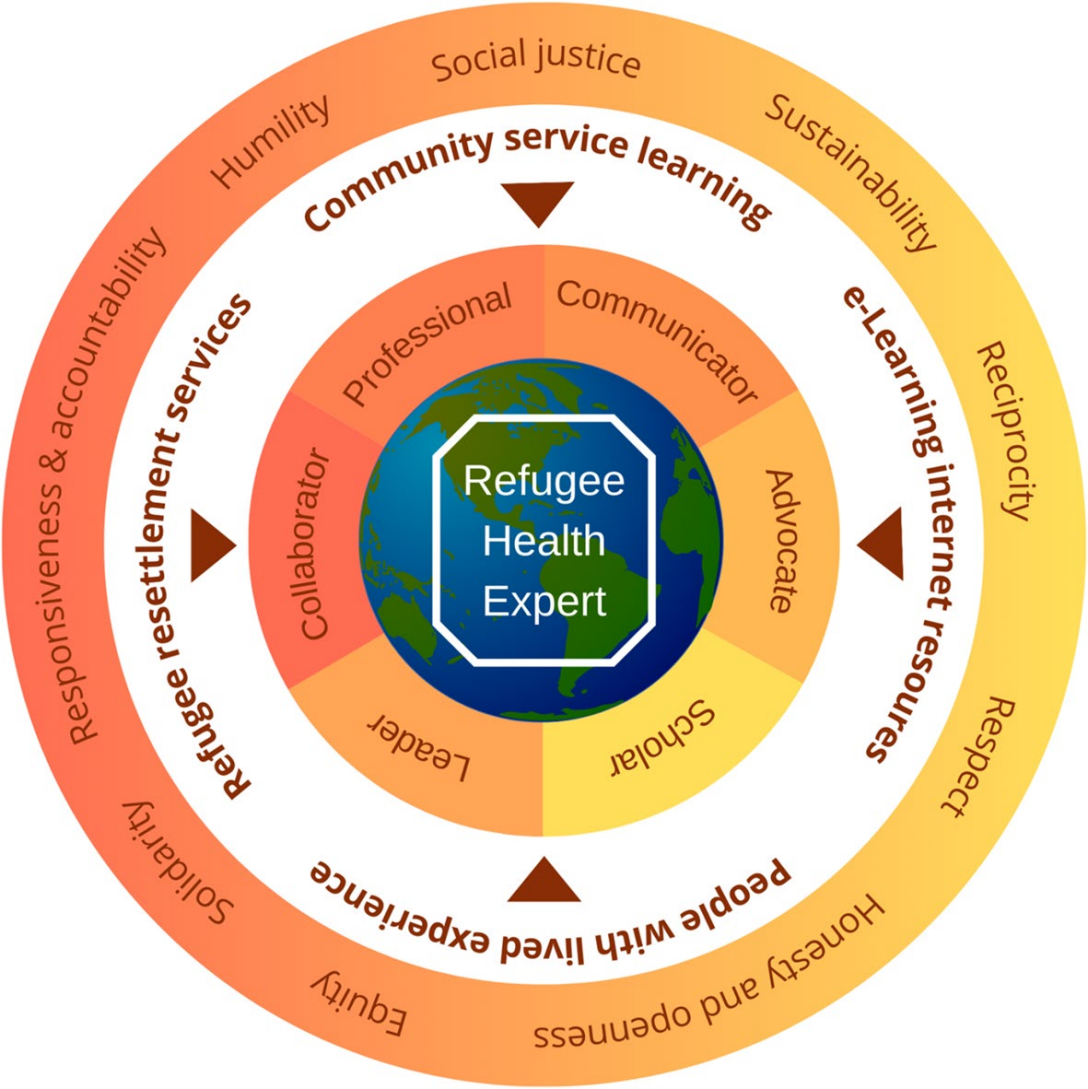
Refugee Health Curriculum Framework. Outer shell: values and principles, inner shell: learning methods, innermost shell: core refugee health competencies (see Table 4)

The proposed primary learning objectives for our refugee and migrant health curriculum framework were based on the CanMEDS 2015 competency based framework[22] and CanMEDS Family Medicine 2017 framework[40]. Our cross cutting learning objectives incorporate many of the topics discussed in the leading refugee and migrant health textbooks[41–43] and published evidence based clinical guidelines for refugees and migrants[1, 44–46]. Similar to existing education frameworks [20, 21], our practical curriculum framework incorporates proposed values and principles, core learning objectives and competencies, and recommended teaching methods (see Fig. 3).

Of the learning objectives identified, the ‘communicator’ competency emerged as a fundamental skill that every student requires to be able to provide effective care for refugees and migrants [9, 47]. Cross-cultural communication in refugee health goes beyond the basic communication skills needed to provide care to Canadian-born patients. For example, a recent four-country study on the use of interpreters with refugee populations in primary care emphasized the importance of using interpreters, but also reports on the ongoing technology, human and time challenges of consistently working with interpreters [48]. Given the high prevalence of trauma in most refugee populations [49], clinical approaches are now integrating more refugee-friendly systems, such as clinical navigators and communication approaches that are included in trauma informed care[1]. The field of medical anthropology and cultural psychiatry have contributed both a better understanding of cultural idioms of distress and global mental health[8] and it is now important to ensure students have an opportunity to explore culturally diverse disease explanatory models within their training.

In addition to the core competency of communication, the ‘advocate’ competency appeared prominently in our findings. Current literature points to the impact of forced migration on mental health, health equity and social determinants of health [24, 50]. Refugee and migrant patients are vulnerable to system inequities and thus require more physician awareness and resources to ensure a successful health equity approach. Teaching advocacy was highlighted by our key informants to be a fundamental requirement to ensure the next generation of physicians understand and are able to implement health equity and interdisciplinary approaches. This conceptual approach will include learning how to use their expertise and influence to be agents of change to improve health outcomes for their refugee patients. Advocacy skills can be enhanced with community service-learning programs, mentorship by more senior students and engaged faculty, as well as taking part in other student-led advocacy initiatives.

This refugee and migrant health framework also identifies methods of curriculum delivery and evaluation to support longitudinal learning. Medical schools have used a wide range of interdisciplinary delivery methods to teach refugee and migrant health. For example, the use of online resources (i.e., e-learning) has become significant due to reduced in-person learning opportunities. Our study also emphasizes the continued importance of community service learning as a practical, hands-on and reflection opportunity to develop cultural safety practices, trauma-informed cross cultural communication skills and the appreciation for interprofessional collaboration and advocacy towards improving refugee and migrant health outcomes [28].

Our findings showed limited formal, well developed evaluation components for refugee health curriculum. Such findings suggest the refugee health curriculum is still early in its development. Robust evaluation metrics could improve the value and outcomes of refugee health curriculum. Our scoping review identified learners self-reported increased communication skills and positive and satisfying experiences. Rashid et al. (2020) also identified that learning outcomes were based primarily on student perception, including more confidence in working with refugee populations, development of advocacy skills and understanding barriers to care [51]. Interdisciplinary community service learning also includes useful reflection exercises. However, ultimately, there needs to be effective metrics and tools to assess whether students have improved skills and are able to apply these skills in practice. The Refugee Health Curriculum Framework, itself, could be used as a quality improvement model to guide schools in the evaluation of their global health curriculum.

### Strengths and Limitations

The scoping review provided the foundation and direction for our interview and survey questions, which in turn informed the development of a robust framework. Our adopted global health values and principles may not be applicable to all countries given the complex nature of migration patterns globally.

The limitations of our framework include the paucity of our findings on evaluation methods for interdisciplinary refugee health curriculum. We were often unable to clearly discern teaching and evaluation approaches when refugee and migrant population content was already embedded within other undergraduate medical education curricula (i.e., infectious diseases or social determinants of health). For example, there was often overlap between other vulnerable populations (i.e., Indigenous populations) due to differing priorities of each medical school. It was possible that medical schools covered topics such as cultural safety, cross cultural communication, equity while working with different vulnerable populations.

## Conclusions

Our mixed-methods undergraduate medical curriculum framework development study takes an in-depth look at existing refugee and migrant health topics, learning objectives, delivery, and evaluation strategies. It was encouraging to find that most medical schools in Canada do already have some mandatory learning objectives related to refugee and migrant health within the undergraduate medical education curricula. We hope enhancing medical school curricula with our guiding framework will increase the likelihood of success in offering interdisciplinary, equitable, effective, and universal healthcare across the world. We recognize that medical schools as well as other health care programs must collaborate and share their work and curriculum resources to allow for greater advocacy and change.

**Table 1:** Educational content present in the various university curriculum

Content description	No. of universities (n = 14)
Epidemiology/demographics of refugees and immigrants new to Canada	11
Barriers refugees and immigrants face when accessing care	13
Challenges and pitfalls of providing care to refugees and immigrants	11
Refugee and immigrant support services in the community	11
Collaborating with allied health, settlement staff and lawyers when providing care to newcomers to Canada	8
Communication skills, cultural and ethical issues when dealing with refugee and immigrant populations (including working with interpreters)	12
Vaccination and screening newly arrived refugees and immigrants for infectious diseases in children and adults	9
Mental health of refugee and immigrant populations (posttraumatic stress disorder (PTSD), depression, adjustment disorders)	9
Reproductive health in refugee and immigrant populations (contraception, pregnancy care, female genital mutilation, intimate partner violence etc.,)	5
Managing chronic non-communicable diseases in refugee and immigrant adults (cancer screening, diabetes screening, cardiovascular disease screening, etc.,)	8
Managing chronic non-communicable diseases in refugee and immigrant children (Oral health, vision care, malnutrition, hereditary anemias, etc.,)	5
Demonstrate basic understanding between health and human rights	7
Social determinants affecting health of refugee populations	12
Being aware of boundary issues that can come up with refugee and vulnerable populations	5

**Table 2:** Methods of content delivery mentioned in the university survey responses

**Methods of content delivery**	**No. of universities (n = 14)**
Large group lectures	12
Small group workshops	7
Electronic/internet tools such as e-learning modules	3
Teaching sessions with standardized patients	2
Portfolio/self-reflection guide	0
Pre-clerkship exposures (settlement agency placements, etc.)	8
Clerkship exposures (core rotations working with refugee or immigrant populations, etc.,)	4

**Table 3:** Primary Learning Topics. By the end of the undergraduate medical training a student will be able to

Understand the importance and need to offer culturally safe and competent healthcare in a trauma informed manner
Communicate effectively across cultures with humility and openness
Explore the issues related to the care of refugees including screening for infectious and chronic illness, prevention and promotion of health including mental health and women’s health
Review the demographics related to refugees and migrant patient populations
Identify the social determinants of health which create barriers for refugees and migrants when accessing health care
Understand the importance of a collaborative team-based approach including being aware of the various support services available to refugees and migrants in the community
Reflect on personal bias and knowledge gaps, while showing respect for cultural and gender diversity of the patient population

**Table 4:** Secondary Learning Topics. In addition to adopting primary topics, medical learners may also be able to

Learn from and work collaboratively with interpreters and settlement workers
Acknowledge challenges in providing care for refugee and migrant populations and continuously work towards overcoming such challenges
Obtain updated information on pertinent information from refugee health and policy, as well as understand how they may impact care
Offer referral services for refugee and migrant families who may require additional counselling and psychological based services
Describe the various resources in the community to support refugees with the aim to improve health outcomes
Understand local vaccination guidelines and approaches for refugees and migrants
Develop an appreciation for how to advocate for refugee clients with letter writing including supporting legal, social and personal needs, including housing, literacy, and citizenship
Gain an understanding of health equity and how system level changes led by socially accountable physicians can lead to improved health outcomes for refugees and migrants
Identify key patient centered factors when reviewing the latest refugee and migrant specific evidence-based guidelines

**Table 5:** Values and Principles to Guide the Curriculum Framework (Redwood Campbell 2009)

Social justice	fair and impartial access to the benefits of society including the right to health
Sustainability	living and working within the limits of available physical, natural and social resources in ways that allow living systems to thrive in perpetuity
Reciprocity	multidirectional sharing and exchange of experience and knowledge among collaborating partners
Respect	for the history, context, values and cultures of communities with whom we engage
Honesty and openness	in planning and implementation of all collaborations
Humility	in recognizing our own values, biases, limitations, and abilities
Responsiveness and accountability	to students and faculty and diverse communities with whom we are involved
Equity	promoting the just distribution of resources and access, especially with respect to marginalized and vulnerable groups
Solidarity	ensuring that objectives are aligned with those of the communities with which we are working

**Table 6:** Refugee Health Competency-Based Learning Objectives. The learner engaged in refugee and migrant health will be able to

Expert	Establish therapeutic, patient centered rapport and understand the importance of delivering comprehensive evidence-based care that is specific to the needs of refugee and migrant populations
Communicator	Communicate with refugee and migrant patient populations and identify student inherent bias’ and address relevant gaps such as language barriers, differing cultural perspectives, and health literacy
	Use a ‘trauma informed care’ approach when addressing disease screening and prevention strategies
Collaborator	Practice a collaborative team-based approach, including establishing positive working relationships with other health care professionals, medical interpreters and community leaders, including legal, religious and cultural representatives
Leader	Describe various trauma informed approaches to improve cultural safety (choice, collaboration, trustworthiness and empowerment), evidence based clinical care and constant quality improvement for refugee and migrant clinical care
Health Advocate	Identify the social determinants of health and barriers to culturally appropriate care affecting refugee and migrant patients
	Describe the various resources in the community to support refugees with the aim to improve health outcomes
	Gain an understanding of health equity and how system level changes led by socially accountable physicians can lead to improved health outcomes for refugees and migrants
Professional	Show respect for, and knowledge of, the demographic and cultural and gender diversity of their patient population
	Reflect on their own bias and knowledge gaps pertaining to the unique needs and barriers refugee and migrant patient populations face when accessing healthcare
Scholar	Identify key patient-centered factors when reviewing the latest refugee and migrant evidence-based clinical prevention guidelines

## Supplementary Information

**Additional file 1:** 

**Additional file 2:** 

**Additional file 3:** 

**Additional file 4:** 

**Additional file 5: **

**Additional file 6: **

**Additional file 7:**

## Data Availability

The datasets generated and analysed during the current study are available in the corresponding author’s OneDrive repository, please email the corresponding author (dgruner@bruyere.org) to receive a link to the de-identified datasets.

## Abbreviations

GRAMMS: Good Reporting of a Mixed Methods Study
TB: Tuberculosis
HIV: Human Immunodeficiency Virus
HCV: Hepatitis C Virus
PTSD: Posttraumatic stress disorder
