# Supplementary material for: An undergraduate medical education framework for refugee and migrant health: Curriculum development and conceptual approaches
Source: BMC Med Educ. 2022 May 16;22:374. doi: 10.1186/s12909-022-03413-8 (PMC9109444; doi:10.1186/s12909-022-03413-8)
Supplement: Supplementary file 1 — Additional file 1: [file 12909_2022_3413_MOESM1_ESM.docx]

**Additional File 1**: Refugee and Migrant Health in Undergraduate Medical Education: A Scoping Review

This scoping review was conducted in order to map existing literature regarding refugee and migrant health education at the undergraduate medical level. More specifically, the primary objective was to identify educational content that has been delivered at the medical undergraduate level regarding refugee and migrant health. Secondary objectives for the review were to identify methods of content delivery and associated educational outcomes. The results were then used to assess common themes emerging from the literature and to assess whether any gaps in knowledge exist in the literature. The methodology for this scoping review followed the approach developed by Arksey and O’Malley (2005) with recommendations made by Levac, Colquhoun and O’Brien (2010).^(1,2)^ The methodology of a scoping review was best suited for our purposes given our broad research objectives and the goal of identifying gaps in current research.

**Literature search**

The literature search was conducted by a health sciences librarian to identify all relevant articles from inception through to September 2018 using the following electronic databases: Medline and Medline in Process (via OVID), Embase Classic + Embase (via OVID), Global Health (CAB Direct), PsycINFO (via Ovid), the Cumulative Index of Allied Health and Nursing Literature (CINAHL via EBSCOHost), ERIC (via Ovid), Education Source (via EBSCOHost) and Best Evidence Medical and Health Professional Education (BEME) database. The search strategy was established through consensus of the team. The search was designed to associate terms related to refugee and migrant health (e.g., asylum seekers, stateless and displaced) or cultural safety (e.g., cultural competency, cultural humility and cultural diversity) with terminology analogous to undergraduate medical education (e.g., medical students, clerkship and pre-clerkship). Given the widespread inclusion of cultural competence in the curricula of medical school and its association with refugee and migrant health,^(3)^ we incorporated cultural competency and associated terms into the search in order to capture relevant articles. We considered the addition of global health and equivalent terms to the search strategy for similar reasons.^(4,5)^ However, we did not include global health in our search terms primarily due to restrictions in feasibility. The search strategy developed for Medline (which served as the basis for translations of the search strategy into other databases) is outlined in Figure 1 below.

**Eligibility criteria**

We included all types of articles that presented a detailed description of an educational intervention at the undergraduate medical level explicitly related to refugee and migrant health. However, at minimum, the publication had to include an abstract. To keep the scoping review focused on refugee and migrant health, we only included articles on potentially related domains, such as cultural competency and global health, if there was a stated connection with the care of displaced populations.

We excluded articles if they were in a language other than English or French, they were published before 1998 or after September 2018, they did not include an organized educational intervention, or they were describing an educational intervention for health care providers other than medical students such as residents, physicians and/or allied health professionals. The cut-off date for publication was established in 1998 to help ensure included articles were still relevant. This range captures articles published since the Liaison Committee on Medical Education set standards in the year 2000 for medical school curricula regarding cultural sensitivity training.^(6)^

**Article Review**

The search results were uploaded to the systematic review software Covidence^(7)^ for screening and review. Most duplicates were removed automatically through Covidence, though some additional duplicates had to be removed manually. Title and abstract screening were performed independently by two reviewers using the pre-determined eligibility criteria. Conflicts between the two reviewers were resolved by a third reviewer. A single reviewer then performed the full-text review for the remaining articles and, using the same pre-established eligibility criteria, determined which articles would be included in the scoping review.

**Charting the data**

Data abstraction was performed by a single reviewer using an iterative data extraction form that was developed by the reviewer and approved by the faculty refugee and migrant health expert. The main characteristics of each article that were extracted include: first author, year of publication, setting (associated university and country), learners (sample size and level(s) of participating medical students), research design, content of educational intervention, method(s) of content delivery, participation/length (mandatory or optional, length of time committed), instructors/facilitators, educational assessment method, educational outcomes and associated Kirkpatrick level of educational outcomes. The four-level Kirkpatrick model of evaluating training programs consists of the following: level one (reaction), level two (learning), level three (behavior) and level four (results).^(8)^

**Data Mapping**

Descriptive statistics were used to summarize information regarding the setting, learners and type of participation. A specific focus was then placed on analyzing the publications for the primary and secondary objectives of the scoping review: identifying the content that has been delivered at the medical undergraduate level regarding refugee and migrant health, the methods of content delivery, and associated educational outcomes. The specific content described in each intervention was summarized into broader categories in order to facilitate comparison between articles using descriptive statistics. For example, articles which described educational content related to tuberculosis, HIV, malaria and/or other tropical diseases were considered to have content related to the category of communicable diseases. A similar approach was used to distill the various described methods of content delivery. Given the heterogeneity in the articles and the lack of quantitative outcomes, we elected not to do a quantitative synthesis of the reported educational outcomes. However, key themes and trends related to the available educational outcomes were qualitatively summarized.

**Figure 1:** Search strategy for Medline database

1. "Emigrants and Immigrants"/

2. Refugees/

3. cultural diversity/

4. "Emigration and Immigration"/

5. Culture Competency/

6. Culturally Competent Care/

7. (refugee* or immigrant* or stateless or emigrant* or displaced or (asyl* adj1 seek*) or (newly

adj arrived) or resettlement).tw.

8. (cultural* adj2 (divers* or safety or competen* or humility or sensitiv*)).tw.

9. or/1-8

10. Clinical Clerkship/

11. education, medical, undergraduate/

12. Students, Medical/

13. (undergraduate* adj2 education*).tw.

14. (clinical adj2 (apprentic* or clerkship*)).tw.

15. (UGME or UME).tw.

16. (preclerkship* or pre-clerkship*).tw.

17. (medical* adj3 student*).tw.

18. or/10-17

19. 9 and 18

**References**

1. Arksey H, O’Malley L. Scoping studies: towards a methodological framework. International journal of social research methodology. 2005;8(1):19–32.

2. Levac D, Colquhoun H, O’Brien KK. Scoping studies: advancing the methodology. Implementation science. 2010;5(1):69.

3. Koehn PH, Swick HM. Medical education for a changing world: moving beyond cultural competence into transnational competence. Academic Medicine. 2006;81(6):548–56.

4. Houpt ER, Pearson RD, Hall TL. Three domains of competency in global health education: recommendations for all medical students. Academic Medicine. 2007;82(3):222–5.

5. Battat R, Seidman G, Chadi N, Chanda MY, Nehme J, Hulme J, et al. Global health competencies and approaches in medical education: a literature review. BMC Medical Education. 2010;10(1):94.

6. Lie D, Boker J, Cleveland E. Using the tool for assessing cultural competence training (TACCT) to measure faculty and medical student perceptions of cultural competence instruction in the first three years of the curriculum. Academic Medicine. 2006;81(6):557–64.

7. Covidence - Better systematic review management [Internet]. Covidence. [cited 2021 Jan 7]. Available from: https://www.covidence.org/

8. Kirkpatrick DL. Techniques for evaluating training programs. Classic writings on instructional technology. 1996;1(192):119.
