# Supplementary material for: An undergraduate medical education framework for refugee and migrant health: Curriculum development and conceptual approaches
Source: BMC Med Educ. 2022 May 16;22:374. doi: 10.1186/s12909-022-03413-8 (PMC9109444; doi:10.1186/s12909-022-03413-8)
Supplement: Supplementary file 2 — Additional file 2: [file 12909_2022_3413_MOESM2_ESM.docx]

**Additional file 1**: Interview Guide

My name is [Name] and I am an academic family physician and clinician teacher in Ottawa. I am also the director of Global Health for undergraduate medical education at the University of Ottawa. We are doing an environmental scan of the current state of refugee and immigrant health education at the undergraduate level in Canada. We hope to learn what other Canadian Medical schools are delivering to their students with respect to refugee and immigrant health curriculum currently. One of the long term goals is to eventually engage experts in refugee health and curriculum content leads in a Delphi like process, with the goal of reaching a consensus on what “should” be part of the curriculum for refugee and immigrant health for undergraduate medical students across Canada.

As an initial step of the environmental scan we are interested in having short telephone discussions with the global health faculty leads or curriculum content leads for refugee health across Canada. Following our discussion, we have a short 3-5 minute online survey to capture some of the topics in the discussion. This project was classified by Ottawa Health Science Network Research Ethics Board as quality improvement. All responses will remain confidential.

1) Is it alright if we record this conversation?

a. If no, then inform interviewee that I will take notes during the discussion.

2) Does your institution currently have a formal undergraduate medical curriculum for refugee and immigrant health?

3) What are some of the broader topics covered in your curriculum?

4) Do you currently have formal learning objectives for this curriculum, is this something you could share as a PDF or word document?

5) Would you be willing to participate in a short 3-5 minute online survey in a few weeks?

6) Do you have any questions for me?
