# Supplementary material for: An undergraduate medical education framework for refugee and migrant health: Curriculum development and conceptual approaches
Source: BMC Med Educ. 2022 May 16;22:374. doi: 10.1186/s12909-022-03413-8 (PMC9109444; doi:10.1186/s12909-022-03413-8)
Supplement: Supplementary file 3 — Additional file 3: [file 12909_2022_3413_MOESM3_ESM.docx]

**Additional file 3:** Online Survey

Thank you for agreeing to participate in this short questionnaire to explore the state of refugee and immigrant health education at the undergraduate level in Canada. This survey should take 3 - 5 minutes to complete. All responses will remain confidential. This project was classified by Ottawa Health Science Network Research Ethics Board as quality improvement. If there are any questions regarding this survey, please contact [NAME + EMAIL].

1. Please indicate the name of your University:

o Dalhousie University

o McGill University

o McMaster University

o Memorial University of Newfoundland

o Northern Ontario School of Medicine

o Queen’s University

o Université de Montréal

o Université Laval

o University of Alberta

o University of British Columbia

o University of Calgary

o University of Manitoba

o University of Ottawa

o University of Saskatchewan

o Université de *Sherbrooke*

o University of Toronto

o University of Western Ontario

#### 2. Within your undergraduate medical curriculum, are there specific objectives that relate to refugee/immigrant/migrant health?

o Yes

o No

3. What does your undergraduate refugee and immigrant health curriculum consist of? (select all applicable)

Curriculum refers to mandatory activities or experiences that students of your institution must complete.

o Large group lectures

o Small group workshops

o Electronic / internet tools such as e-learning modules

o Teaching sessions with standardized patients

o Portfolio / self-reflection guide

o Pre-clerkship exposures (settlement agency placements, etc.,)

o Clerkship exposures (core rotations working with refugee or immigrant populations, etc.,)

o Other (please specify):

4. Over the entire undergraduate medical program at your institution, how many hours do you estimate your students spend learning refugee and immigrant health?

o 0 – 5 hours

o 5 – 10 hours

o 10 – 15 hours

o 15 – 20 hours

o >20 hours

5. Which of these topics are currently covered in your refugee and immigrant health curriculum? (select all applicable)

1) Epidemiology/demographics of refugees and immigrants new to Canada

2) Barriers refugees and immigrants face when accessing care

3) Challenges and pitfalls of providing care to refugees and immigrants

4) Refugee and immigrant support services in the community

5) Collaborating with allied health, settlement staff and lawyers when providing care to newcomers to Canada

6) Communication skills, cultural and ethical issues when dealing with refugee and immigrant populations (including working with interpreters)

7) Vaccination and screening newly arrived refugees and immigrants for infectious diseases in children and adults

8) Mental health of refugee and immigrant populations (PTSD, depression, adjustment disorders)

9) Reproductive health in refugee and immigrant populations (contraception, pregnancy care, female genital mutilation, intimate partner violence etc.,)

10) Managing chronic non-communicable diseases in refugee and immigrant adults (cancer screening, diabetes screening, cardiovascular disease screening, etc.,)

11) Managing chronic non-communicable diseases in refugee and immigrant children (Oral health, vision care, malnutrition, hereditary anemias, etc.,)

12) Demonstrate basic understanding between health and human rights

13) Social determinants affecting health of refugee populations

14) Being aware of boundary setting with refugee and vulnerable populations.

15) Other: please specify

6. Does your institution have specific and mandatory learning objectives regarding refugee and immigrant health?

o Yes

o No

7. Please paste here your learning objectives regarding refugee and immigrant health, or a link to where the learning objectives could be found.

END OF SURVEY – THANK YOU FOR PARTICIPATING
