# Supplementary material for: An undergraduate medical education framework for refugee and migrant health: Curriculum development and conceptual approaches
Source: BMC Med Educ. 2022 May 16;22:374. doi: 10.1186/s12909-022-03413-8 (PMC9109444; doi:10.1186/s12909-022-03413-8)

**Additional file 4**: PRISMA flow diagram of literature search and study selection for scoping review.


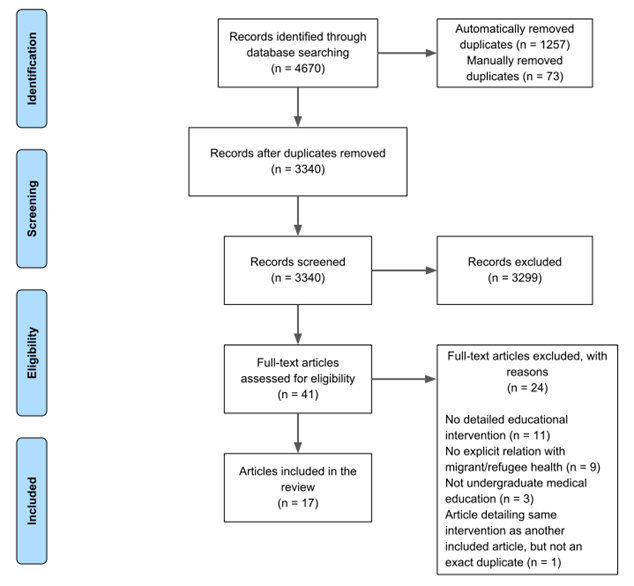

Supplement: Supplementary file 4 — Additional file 4: [file 12909_2022_3413_MOESM4_ESM.docx]
