# Supplementary material for: An undergraduate medical education framework for refugee and migrant health: Curriculum development and conceptual approaches
Source: BMC Med Educ. 2022 May 16;22:374. doi: 10.1186/s12909-022-03413-8 (PMC9109444; doi:10.1186/s12909-022-03413-8)
Supplement: Supplementary file 5 — Additional file 5: [file 12909_2022_3413_MOESM5_ESM.docx]

**Additional file 5**: Scoping review results

Seventeen articles met our eligibility criteria and were included in this scoping review. The most prevalent topics from the scoping review included cultural safety and cross-cultural communication,(1–7) working with interpreters,(1,3,8–12) clinical experience with refugee/migrant patients,(3,6,10–14) refugee and migrant law and health policies,(2,4,8,14–16) as well as disease screening, prevention and immunization(1,4–6,10,14). Content delivery methods most commonly used included experiential and community service learning,(1,3,5,6,8–14,16) but other methods included didactic teaching,(1,2,5,7–9,14,15) group and cased based learning,(1,2,5,7–9,15,16) and interactive seminars as well as panels(1,2,4,6,12,14,15). Articles reporting on educational outcomes and evaluation strategies were rare, however learners self-report increased cross-cultural knowledge and communication skills and generally reported positive and satisfying experiences. See Additional file 5 for full results of the scoping review. **Characteristics of articles and educational interventions**
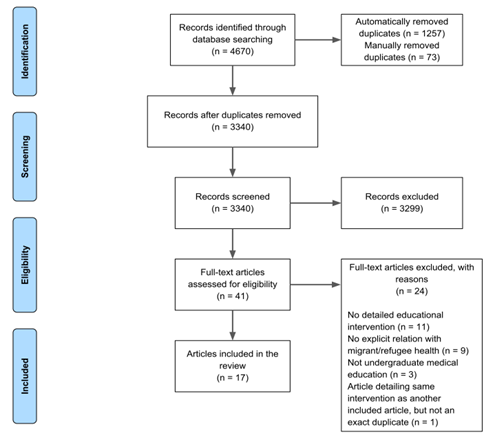
Of the 3340 records that were identified following duplicate removal, 41 publications underwent full-text review, and 17 articles met eligibility criteria for the scoping review (Figure 1). Most of these articles originated from the United States (8 articles, 47%)(1–3,5,13,14,16,17) and Canada (4 articles, 24%),(6,10–12) and all remaining articles came from other developed countries.(4,7,9,15,18) Medical student learners were most commonly in Year 1 and Year 2, though 29% of the articles(5,11,14,16,18) did not specify the level of the medical student participants. Most of the educational interventions were optional (12 articles, 71%)(1–6,8,10,12,14,15,18) while only two (11%)(7,9) were either mandatory or mandatory with optional electives. Table 1 summarizes the descriptive statistics regarding setting, learners and participation. The summary of all included articles, as guided by the data abstraction form, is available in Figure 2.

**Figure 1** PRISMA flow diagram of literature search and study selection

**Table 1** Setting, learner and participation characteristics of 17 included educational interventions

| **Country** | | **No. of articles (% of 17)** |
| --- | --- | --- |
| **United States**(1–3,5,13,14,16,17) | | 8 (47) |
| **Canada**(6,10–12) | | 4 (24) |
| **United Kingdom**(7,15,18) | | 3 (18) |
| **Australia**(4) | | 1 (6) |
| **Switzerland**(9) | 1 (6) | |
| **Year of medical student learners** | | **No. of articles (% of 17)** |
| **Year 1**(2,3,6,8–10,12,13) | | 8 (47) |
| **Year 2**(2,3,6,8,10,13) | | 6 (35) |
| **Year 3**(1,3,8,9,15) | | 5 (29) |
| **Year 4**(3,4,7–9) | | 5 (29) |
| **Year 5**(4) | | 1 (6) |
| **Year 6**(4,9) | | 2 (12) |
| **Not stated**(5,11,14,16,18) | | 5 (29) |
| **Participation** | | **No. of articles (% of 17)** |
| **Optional**(1–6,8,10,12,14,15,18) | | 12 (71) |
| **Not stated**(11,13,16) | | 3 (18) |
| **Mandatory**(7) | | 1 (6) |
| **Mandatory with optional electives**(9) | | 1 (6) |

**Figure 2:** Characteristics of 17 Educational Interventions on Refugee and migrant Health at the Medical Undergraduate Level

| **First author, year** | **Setting** | **Learners (sample)** | **Research design** | **Educational content** | **Methods of delivery** | **Participation/length** | **Instructors/facilitators** | **Educational assessment method** | **Kirkpatrick level** | **Educational outcomes** |
| --- | --- | --- | --- | --- | --- | --- | --- | --- | --- | --- |
| **Albritton,**  **2002**(13) | Medical College of Georgia,  United States | Medical students, Years 1-2 | Not applicable | Participation in migrant health clinic with nursing and allied health students | Experiential learning | Not stated | Not stated | Not stated | Not applicable | Not stated |
| **Griswold,**  **2003**(14) | State University of New York at Buffalo,  United States | Medical students (~50), Year(s) not stated | Not applicable | Participation in Refugee Health Night program (meeting with preceptors and refugee health clinic) | Problem-based cases, experiential learning followed by debrief | Optional,  Each Refugee Health Night approx. 4 hours | 2 family medicine preceptors experienced working with refugees and/or in 3^rd^ world countries | No formal assessment (verbal process evaluations and written comments) | Level 1 | Students learned about cultural issues, developed communication skills and reported positive learning experiences through their contact with refugees. |
| **Griswold,**  **2006**^(3)^ | State University of New York at Buffalo,  United States | Medical students (133), Years 1-4 (75% year 1, 16% year 2, 9% years 3-4) | Pre/post | Lecture on refugee status and health, cultural lesson, instruction in interpretation methods, participation in refugee evening clinic, various learning activities (storytelling sessions by refugees, sessions on screening/immunization, educational sessions on legal issues, tuberculosis, mental health, female care, diabetes, and heart disease) | Experiential learning, lectures, refugee panels (optional), student presentations (optional) | Optional, Participation in minimum of 2 evening clinics (time not specified), various learning activities between 1-2 hours long | Family doctor preceptors with experience in international medical settings, case managers with refugee settlement agency | Pre- and post- programme surveys (Cultural Awareness Self-Assessment Questionnaire, 17 items, 5-point Likert scale survey) | Level 1 | Students reported significant improvement in the 3 domains measured by the questionnaire: knowledge of psychosocial issues, knowledge of cultural issues and communication skills. |
| **Broome,**  **2007**(18) | University of Leeds,  United Kingdom | Medical students, Year(s) not stated | Not applicable | Intercalated BSc in International Health with modules on health systems, health policy and development, communicable disease, non-communicable disease, maternal/child/reproductive health, research methods, literature review project and research product | Role-playing exercises, research project | Optional | Not stated | No formal assessment, writers report their experience in this program as student participants | Level 1 | Participants report better understanding the need for cost-effective and sustainable interventions, gaining practical experience in health policy development and gaining skills to practice evidence-based medicine |
| **Pottie,**  **2007**(6) | University of Ottawa,  Canada | Medical students (45),  Years 1-2 | Post only | Training in refugee health and cultural sensitivity workshop, prevention outreach program (students paired with newly arrived refugee family, perform a medical history and debriefing workshop) | Internet-based training module, workshops, family physician mentorship, experiential learning | Optional | Family physician teachers and medical faculty with expertise in refugee health | Semi-structured follow-up interview with 5 medical students after program pilot test | Level 1 | Students reported improvement in cross-cultural knowledge and skills, but were at time disheartened by refugees’ poor access to primary care |
| **Dussan,**  **2009**(2) | Michigan State University,  United States | Medical students (42),  Years 1-2 | Quasi-experimental (comparison group) | Topics addressed include overview of displaced persons, outreach agencies for refugees, refugee mental health, legal aspects of refugee care, common refugee healthcare issues, providing culturally appropriate care, sociological perspectives on refugee care | Lectures, discussion sessions, refugee discussion panel, arts (documentary viewing, film viewing, and book reading) | Optional,  1-hour weekly course (unspecified total number of classes) | Local experts in refugee advocacy and care (including professors, community physicians and social workers) | Pre- and post-questionnaires (20 items, of which 14 were 5-point Likert scale reflecting attitudes and beliefs) given to all pre-clinical students. Pre- and post-questionnaires of non-participant students served as controls for participants students | Level 1 | Participants reported statistically significant (p < 0.05) change in multiple attitudes/beliefs items including knowledge of refugee medical and mental health issues and identifying cultural issues compared to peers. 97.6% of participants anticipated working with refugees in the future. |
| **Hill,**  **2009**(15) | Brighton Sussex Medical School,  United Kingdom | Medical students,  Year 3 | Not applicable | Interprofessional program on vulnerable populations with a focus on refugees and asylum seekers. Themes included refugee legal and policy context, social work perspectives, service user/carer perspectives and creative practice | Lectures, small group discussions, Q&A session with asylum seeker, inter-professional group work | Optional,  Multiple 3-hour sessions over 8 weeks | Faculty with experience in working with disadvantaged groups, service user (asylum seeker) facilitators | Feedback from participants (methods not stated) | Level 1 | Overall, students reported a positive experience and highlighted the value in gaining insight from service users and the importance of value/knowledge integration. |
| **Spencer,**  **2010**(7) | Newcastle University Faculty of Medical Sciences,  United Kingdom | Medical students (719),  Year 4 | Post only | Session on asylum seekers and refugees based on a cultural competence training workshop | Lectures, group work, discussions | Mandatory,  2-hour session | Co-facilitation by senior medical student or junior doctor and asylum seeker from community | Post-questionnaires (Items includes Likert scales and free text, but questionnaire content not otherwise specified) | Level 1 | Participants gave positive feedback, reported better understanding of the difficulties that face refugees and found session eye-opening |
| **Gagnon.**  **2011**(11) | Laval University,  Canada | Medical students,  Year(s) not stated | Not applicable | Longitudinal rotation where students follow 2 refugee families and participate in interprofessional work (including interpreters) at a refugee clinic and collaborate with a community organization | Experiential learning, written reflection and a presentation | Not stated,  Longitudinal rotation over 2 month period | Not stated | Feedback from participants (methods not stated) | Level 1 | Participants reported a high degree of satisfaction with this rotation |
| **Laven,**  **2011**(4) | University of Adelaide Medical School,  Australia | Medical students,  Years 4-6 (~2/3 in year 6, remainder in year 4-5) | Not applicable | Course covers 3 domains of burden of global disease, travelers medicine and immigrant health. Specific topics include communicable diseases, geriatric medicine, women’s health, mental health, child health, oral health, law/politics, Indigenous health and cultural diversity. | Interactive seminars, group work and presentation | Optional,  Total of 40 hours over 3 weeks | Professionals practicing in International  Health in a variety of disciplines (including  engineering, nursing, politics, dentistry and  general practice) | Qualitative evaluations from students (methods not stated) | Level 1 | Student opinion regarding the course was largely positive with some supporting that it should be a core component in medical education |
| **Asgary,**  **2013**(8) | Mount Sinai School of Medicine,  United States | Medical students (50),  Years 1-4 (27% year 1, 13% year 2, 12% year 3, 7% year 4) | Pre/post | Introductory workshop with teaching on asylum law, medical issues in torture victims, asylum seeker evaluation and affidavit writing.  Students participated in clinical session(s) of asylum seeker evaluations (including working with interpreters) and supervised affidavit writing. | Lectures, discussions, case presentation, experiential learning, supervised affidavit writing | Optional,  Introductory workshop and at least one clinical session (hours not stated) | Faculty preceptor with extensive experience working with refugees and internationally and with formal training in asylum seeker evaluation and torture documentation | Pre- and post- curriculum surveys (developed 32 item survey to evaluate knowledge of 5 domains regarding care of torture victims) | Level 2 | Statistically significant (p < 0.05) improvement in knowledge and attitudes in several domains including physical and psychological sequelae of torture, legal aspects of seeking asylum in the US and attitudes toward working with torture survivors. 79% reported increased interest in future involvement in human rights issues. |
| **Palmer,**  **2014**(5) | Oregon Health & Science University,  United States | Medical students (68),  Year(s) not stated | Not applicable | Course includes training on common medical issues in migrants (including diabetes, mental health, nutrition and oral health) and cross-cultural communication. Students participate in interprofessional teams (including working with interpreters) providing health screening and health guidance to foreign born clients | Arts (exhibit visit), lectures, discussions,  experiential learning, written reflection | Optional,  Introductory session and 4 biweekly 5-hour community sessions | Faculty members from various health professions | End-of-course mandatory written reflections were analyzed for major themes | Level 1 | The participants reported gaining cross-cultural knowledge, developing skills in communication, learning to work in multidisciplinary team, increased understanding of illness context and enjoying learning from clients’ experiences |
| **Warmington, 2014**(12) | University of Ottawa,  Canada | Medical students (46),  Year 1 | Not applicable | Participation in Refugee Health Initiative where students are matched to a newly arrived refugee family and perform a medical intake interview as well as volunteer in activities such as a Community Health Fair. Students work with community partners (including interpreters) and receive training sessions in global health. | Training sessions, experiential learning | Optional,  Some students participated to complete program-required 30 hours of community service | Community case managers and medical professionals with expertise in global health | Feedback from participants (methods not stated) | Level 1 | Students reported increased cultural competency, knowledge about refugee/migrant populations and comfort working with vulnerable populations |
| **Bertelsen, 2015**(1) | New York University School of Medicine,  United States | Medical students (33),  Majority in year 3 | Post only | Course content includes clinical assignments with diverse population, cultural competency training, patient case discussions (tropical medicine, maternal health, tuberculosis, hypertension, diabetes, working with interpreters, trauma from torture), microbiology workshop on malaria, Global Health journal club, various conferences (infectious diseases and pulmonary), and lectures (topics include global burden disease, health systems, telemedicine, vaccine programs and child health metrics) | Experiential learning, clinical skills simulations, case discussions, interactive workshop, journal club, lectures | Optional,  4 week selective | At least 30 faculty members from 11 different departments (infectious diseases staff represented 29-33% of total number of involved faculty/fellows) | Post-selective electronic survey (mix of quantitative and qualitative assessment questions) | Level 1 | 86% of participants rated selective as excellent, case discussions and clinical skills simulations were rated the highest in terms of learning activities, and students reported the selective contributed the most to their abilities to understand tropical diseases and use cross-cultural communication skills |
| **Casillas, 2015** (9) | Lausanne University,  Switzerland | Medical students,  Years 1, 3, 4, 6 | Not applicable | Training included medical anthropology material in human and social science curriculum, case-based training and sessions on health and migration, communication with an interpreter, mental health, HIV, and public health. Optional 12-week health disparities elective in years 3-4, and 1-month elective rotation in ambulatory clinic with health disparities project in year 6. | Lectures, case discussions, experiential learning (optional), health disparities project (optional) | Mandatory and optional electives,  4 x 2-hour lectures (year 1), 3 x training sessions (year 3), 12-week elective (years 3-4), 1 month elective (year 6) | Not stated | Not stated | Not applicable | Not stated |
| **Duke,**  **2015**(10) | Memorial University of Newfoundland,  Canada | Medical students (477),  Years 1-2 | Not applicable | MUN-MED Gateway Project pairs medical students with newly arrived refugees to conduct medical interviews, health screening, administer tuberculin skin tests and start health initiatives (such as a refugee well-woman pilot project) on a volunteer basis. Students work with multidisciplinary team (interpreters, settlement health worker, public health nurse). | Experiential learning | Optional | Family physician faculty advisor, public health nurse | Not stated | Not applicable | Not stated |
| **McKenzie,**  **2017**(16) | Yale School of Medicine and Georgetown School of Medicine,  United States | Medical students,  Year(s) not stated | Not applicable | Yale University:  Observation of asylum seekers evaluation, introductory literature about asylum medicine with redacted affidavit from the client along with pre- and post- counseling and discussion. There is possibility of performing asylum seeker evaluations and preparing affidavits.  Georgetown University:  Training to perform medical evaluations for asylum seekers in the US. There is an associated curriculum on refugee and asylum health including trauma-informed care.  Both programs have training on asylum law | Observation, discussion, experiential learning (optional), affidavit writing (optional) | Not stated | Yale University:  Not stated  Georgetown University:  Faculty advisor and Physicians for Human Rights | Not stated | Not applicable | Not stated |

**Content**

The specific content described in the educational interventions related to refugee and migrant health was heterogeneous. The categories of content that were most prevalent across the interventions were cultural sensitivity and safety training,(1–7) experience in migrant/refugee health clinic,(3,6,10–14) and working with interpreters(1,3,8–12) (all 7 articles, 41%). This was followed by refugee and migrant law and health policies(2–4,8,15,16) and screening/prevention/immunization(1,3–6,10) (all 6 articles, 35%). Content focusing on specifics of health such as communicable disease (5 articles, 29%),(1,3,4,9,18) mental health (5 articles, 29%),(2–5,9) non-communicable disease (5 articles, 29%),(1,3–5,18) women’s health (4 articles, 24%),(1,3,4,18) child health (3 articles, 18%),(1,4,18) and trauma-informed care (3 articles, 18%)(1,8,16) were less commonly described. Various other categories of reported content included public/population health (5 articles, 29%),(1,2,4,9,18) sociological aspects (3 articles, 18%),(2,9,15) affidavit writing (2 articles, 12%),(8,16) and asylum seeker evaluation (2 articles, 12%).(8,16) Table 2 summarizes the educational content described in the 17 included educational interventions.

**Table 2** Educational content described in 17 included educational interventions

| **Content description** | **No. of articles (% of 17)** |
| --- | --- |
| Cultural sensitivity and safety training(1–7) | 7 (41) |
| Experience in migrant/refugee health clinic(3,6,10–14) | 7 (41) |
| Working with interpreters(1,3,8–12) | 7 (41) |
| Refugee/migrant law and health policies(2–4,8,15,16) | 6 (35) |
| Screening/prevention/immunization(1,3–6,10) | 6 (35) |
| Communicable diseases(1,3,4,9,18) | 5 (29) |
| Mental health(2–5,9) | 5 (29) |
| Non-communicable diseases(1,3–5,18) | 5 (29) |
| **Public/population health**(1,2,4,9,18) | 5 (29) |
| **Women’s health**(1,3,4,18) | 4 (24) |
| **Child health**(1,4,18) | 3 (18) |
| Sociological aspects(2,9,15) | 3 (18) |
| **Trauma-informed care**(1,8,16) | 3 (18) |
| **Affidavit writing**(8,16) | 2 (12) |
| **Asylum seeker evaluation**(8,16) | 2 (12) |

**Methods of delivery**

The most commonly used method of content delivery was experiential learning (12 articles, 71%). (1,3,5,6,8–14,16) Didactic teaching in the form of lectures was the next most described method of delivery (8 articles, 47%). (1–3,5,7–9,15) Interactive forms of content delivery through group discussions (6 articles, 35%), (2,5,7,8,15,16) case-based learning (4 articles, 24%), (1,8,9,14) workshops/interactive seminars (4 articles, 24%), (1,4,6,12) group work (3 articles, 18%), (4,7,15) and refugee panels (3, articles, 18%) (2,3,15) were also used. A variety of other methods of delivery were reported including student presentation (3 articles, 18%), (3,4,11) supervised affidavit writing (2 articles, 12%), (8,16) arts (2 articles, 12%), (2,5) research projects (2 articles, 12%), (9,18) and written reflection (2 articles, 12%). (5,11) Clinical simulation, (1) internet-based module, (6) journal club, (1) direct observation, (16) and role playing (18) were all methods of delivery only reported in a single article (6%). Table 3 summarizes the methods of delivery described in the 17 included educational interventions.

**Educational outcomes**

Many articles either did not provide any educational outcomes or reported only select qualitative feedback from participants without giving a detailed description of assessment methods. Common themes found in the described qualitative and quantitative feedback included positive and/or satisfying learning experiences (1,4,5,7,11,14,15) and learners self-reporting improved cross-cultural knowledge and/or communication skills. (1–3,5,6,12,14) Of note, only one research article had a quasi-experimental study design with a comparison group. (2) As well, only one study provided educational outcomes equivalent to a Kirkpatrick level 2 or higher; the authors assessed participants’ knowledge in five domains regarding the care of torture victims using pre- and post-curriculum surveys. (8)

**Table 3** Methods of content delivery described in 17 included educational interventions

| **Methods of content delivery** | **No. of articles (% of 17)** |
| --- | --- |
| Experiential learning(1,3,5,6,8–14,16) | 12 (71) |
| **Lectures**(1–3,5,7–9,15) | 8 (47) |
| **Group discussions**(2,5,7,8,15,16) | 6 (35) |
| **Case-based learning**(1,8,9,14) | 4 (24) |
| **Workshops/interactive seminars**(1,4,6,12) | 4 (24) |
| **Group work**(4,7,15) | 3 (18) |
| **Refugee panel**(2,3,15) | 3 (18) |
| **Student presentation**(3,4,11) | 3 (18) |
| **Supervised affidavit writing**(8,16) | 2 (12) |
| **Arts (films, books, exhibits)**(2,5) | 2 (12) |
| **Research project**(5,11) | 2 (12) |
| **Written reflection**(5,11) | 2 (12) |
| **Clinical simulation**(1) | 1 (6) |
| **Direct observation**(16) | 1 (6) |
| **Internet-based module**(6) | 1 (6) |
| **Journal club**(1) | 1 (6) |
| **Role playing** exercise(18) | 1 (6) |

**References**

1. Bertelsen NS, DallaPiazza M, Hopkins MA, Ogedegbe G. Teaching global health with simulations and case discussions in a medical student selective. Globalization and health. 2015;11(1):1–8.

2. Dussán KB, Galbraith EM, Grzybowski M, Vautaw BM, Murray L, Eagle KA. Effects of a refugee elective on medical student perceptions. BMC medical education. 2009;9(1):1–8.

3. Griswold K, Kernan JB, Servoss TJ, Saad FG, Wagner CM, Zayas LE. Refugees and medical student training: results of a programme in primary care. Medical education. 2006;40(7):697–703.

4. Laven G, Newbury JW. Global health education for medical undergraduates. Rural and remote health. 2011;11(2):268.

5. Palmer VS, Mazumder R, Spencer PS. Interprofessional global health education in a cosmopolitan community of North America: the iCHEE experience. Academic Medicine. 2014;89(8):1149–52.

6. Pottie K, Hostland S. Health advocacy for refugees: medical student primer for competence in cultural matters and global health. Canadian Family Physician. 2007;53(11):1923–6.

7. Spencer J, McNulty A, Brice A. Providing Care for ‘hard to reach out to’ Patient Groups. Medical Education. 2010;44(3):52.

8. Asgary R, Saenger P, Jophlin L, Burnett DC. Domestic global health: a curriculum teaching medical students to evaluate refugee asylum seekers and torture survivors. Teaching and learning in medicine. 2013;25(4):348–57.

9. Casillas A, Paroz S, Dory E, Vu F, Bodenmann P. Building the diversity bridge abroad: The strategy to implement pre-graduate cultural competency medical education in Lausanne, Switzerland. In Springer, New York, NY 10013 USA; 2014. p. S509–S509.

10. Duke P, Brunger F. The MUN Med Gateway Project: marrying medical education and social accountability. Canadian Family Physician. 2015;61(2):e81–7.

11. Gagnon S. Interdisciplinary Rotation in Refugee Health for Undergraduate Students at Laval University with the Collaboration of a Community Organism: To Innovate to Sensibilize Physicians of Tomorrow to the Reality of Refugees just after their arrival in Canada: OP-107. Medical Education. 2011;45(1).

12. Warmington R, Sickand M, Saliba L, Snyder E, Martel N, Farren-Dai L, et al. Global health education locally: A community service-learning program to support refugees, engage medical students, and fill a gap in the community. Annals of Global Health. 2014;80(3).

13. Albritton TA, Wagner PJ. Linking cultural competency and community service: a partnership between students, faculty, and the community. Academic medicine: journal of the Association of American Medical Colleges. 2002;77(7):738–9.

14. Griswold KS. Refugee health and medical student training. FAMILY MEDICINE-KANSAS CITY-. 2003;35(9):649–54.

15. Hill L, Gray R, Stroud J, Chiripanyanga S. Inter‐professional learning to prepare medical and social work students for practice with refugees and asylum seekers. Social Work Education. 2009;28(3):298–308.

16. McKenzie KC, Mishori R, Tajeda S. Engaging students in the evaluation of asylum seekers: Building capacity, teaching service and resilience. In Springer, New York, NY 10013 USA; 2017. p. S674–S674.

17. Asgary R, Segar N. Barriers to health care access among refugee asylum seekers. Journal of Health Care for the Poor and Underserved. 2011;22(2):506–22.

18. Broome JL, Gordon JK, Victory FL, Clarke LA, Goldstein DA, Emmel ND. International health in medical education: students’ experiences and views. Journal of health organization and management. 2007;
