# Supplementary material for: An undergraduate medical education framework for refugee and migrant health: Curriculum development and conceptual approaches
Source: BMC Med Educ. 2022 May 16;22:374. doi: 10.1186/s12909-022-03413-8 (PMC9109444; doi:10.1186/s12909-022-03413-8)
Supplement: Supplementary file 6 — Additional file 6: [file 12909_2022_3413_MOESM6_ESM.docx]

**Additional file 6**: Significant quotations from key informant interviews

| **Theme** | **Quotation** |
| --- | --- |
| Refugee and migrant health learning objectives | “Presented what challenges… a refugee family might face in the first year. Talked about refugee health, role of settlement agencies, what kind of resources available here but also an introduction to specific refugee health screening, chronic diseases, infectious diseases, but it was very basic for first year.” – *Interview #3* |
|  | “The idea is, the approach has been these are special populations but they should be part of your core practice because you will come across them…We say that you will encounter this. You have this knowledge from the beginning.” –*interview #6* |
|  | “I know that there are pieces embedded in other courses that include patients who are refugees, that include patients who are immigrants, as another factor in you know, here's a cardiology case, here’s a MSK case”- *Interview #2* |
| Active teaching methods | “And one of the things that is, I think a really positive aspect of our community service learning is that …they are meeting up with a … medical student who has…not as much… medical knowledge as a fully qualified physician, but it is… an opportunity for them to liaise with the medical community early on, and oftentimes, as a very positive interaction”*-Interview #9* |
| Overlap with other underserved populations | “There's a lot of foundational content that's relevant to multiple vulnerable populations, for example, multiple communities that needs special attention and special considerations in terms of health care. … So all of those other foundational ones aren't necessarily tagged as refugee health, but they're very relevant when they come to either session.” – *Interview #2* |
|  | “Social equity, social determinants of health, that's a broader topic… here a lot of the focus [is] on Aboriginal community, yes, and that the health deficits there… that probably takes priority.” – *Interview #12* |
|  | “Always question why should this be mandatory over other populations that are similarly vulnerable. This is how people get pitted against each other especially when there is limited time.” – *Interview #7* |
| Challenges of implementing a refugee and migrant health curricula within undergraduate medical education | “So the formal curriculum is very limited, as you're aware, it's such a fight to get anything into the curriculum.” – *Interview #13* |
|  | **“**Yeah, absolutely. And, and the students pick up on, you know, if it's, if it's not essential for the exams, they can be more dismissive of, of the sessions.” – *Interview #12* |
| Value of sharing educational resources across Canadian medical schools | “I would love to expand on refugee health teaching. Why start from scratch when things already exist. If other universities have good resources would be delighted to access and piggyback those resources.”-*Interview #3* |
