# Supplementary material for: An undergraduate medical education framework for refugee and migrant health: Curriculum development and conceptual approaches
Source: BMC Med Educ. 2022 May 16;22:374. doi: 10.1186/s12909-022-03413-8 (PMC9109444; doi:10.1186/s12909-022-03413-8)
Supplement: Supplementary file 7 — Additional file 7: [file 12909_2022_3413_MOESM7_ESM.docx]

**Additional file 7:** Refugee Health Curriculum e-Surveys

A follow-up e-survey provided details related to the refugee health curriculum at 14 of the 17 medical schools. The survey took an average of 3 minutes to complete. We used fourteen* survey responses in the final analysis (response rate: 82.4%). Thirteen surveys were completed in English and one in French. Moreover, five surveys came from Western Canada, four from Ontario, three from Quebec and two from Eastern Canada. The majority of respondents reported having mandatory refugee health learning objectives (8/14). The most prevalent learning objectives included access to care barriers (13/14), social determinants of health for refugees (12/14), cross-cultural communication skills (12/14), global health disease epidemiology (11/14), challenges and pitfalls of providing care (11/14) and mental health (9/14).

* NB: Queens did the interview but not the survey. Laval did the survey but not the interview.
